# Adenosine‐5′‐triphosphate suppresses proliferation and migration capacity of human endometrial stem cells

**DOI:** 10.1111/jcmm.15115

**Published:** 2020-03-09

**Authors:** Svetlana Semenova, Alla Shatrova, Irina Vassilieva, Margarita Shamatova, Natalja Pugovkina, Yuri Negulyaev

**Affiliations:** ^1^ Institute of Cytology of the Russian Academy of Science Saint‐Petersburg Russia

**Keywords:** Ca^2+^ influx, cell cycle, cell migration, endometrial stem cells, extracellular ATP, receptor P2X_7_

## Abstract

Extracellular ATP through the activation of the P2X and P2Y purinergic receptors affects the migration, proliferation and differentiation of many types of cells, including stem cells. High plasticity, low immunogenicity and immunomodulation ability of mesenchymal stem cells derived from human endometrium (eMSCs) allow them to be considered a prominent tool for regenerative medicine. Here, we examined the role of ATP in the proliferation and migration of human eMSCs. Using a wound healing assay, we showed that ATP‐induced activation of purinergic receptors suppressed the migration ability of eMSCs. We found the expression of one of the ATP receptors, the P2X_7_ receptor in eMSCs. In spite of this, cell activation with specific P2X_7_ receptor agonist, BzATP did not significantly affect the cell migration. The allosteric P2X_7_ receptor inhibitor, AZ10606120 also did not prevent ATP‐induced inhibition of cell migration, confirming that inhibition occurs without P2X_7_ receptor involvement. Flow cytometry analysis showed that high concentrations of ATP did not have a cytotoxic effect on eMSCs. At the same time, ATP induced the cell cycle arrest, suppressed the proliferative and migration capacity of eMSCs and therefore could affect the regenerative potential of these cells.

## 1 INTRODUCTION

Mesenchymal stem cells (MSCs) are derived from a wide range of adult tissues, including adipose tissue, cartilage, tendons, tooth pulp and periodontal ligament. By now the most common sources are bone marrow^1^ and adipose tissue.^2^ In recent years, a significant number of publications have appeared on the isolation of mesenchymal cells from the human endometrium. The important role of stem cells in the cyclic regeneration of the endometrium was discovered many years ago.^3^, ^4^, ^5^ However, the population of endometrial stem cells (eMSCs) was first isolated from endometrial tissue samples and characterized only in 2004.^6^, ^7^ Later, researchers isolated and characterized stem cells from menstrual blood samples.^8^, ^9^, ^10^ Endometrium cells are broadly represented in the menstrual blood after desquamation. Even small blood samples are enough to isolate eMSCs.

Endometrial stem cells have a fibroblast‐like morphology typical for all MSCs and represent a heterogeneous cell population consisting mainly of stromal and epithelial cells of the endometrial gland. It has been shown that eMSCs can be differentiated into cells of the tissues of the mesodermal (myocytes, cardiomyocytes, osteocytes, adipocytes, etc) and ectodermal (neurons) rows.^11^ Together with the high availability of eMSCs, their key properties (high proliferative activity, genome stability, high level of plasticity) make these cells a very valuable substrate of cell therapy for many diseases.

Adenosine‐5′‐triphosphate (ATP) is an important multifunctional nucleotide serving as intracellular energy source. However, once outside the cell, it acts as signalling molecule, binding to purinergic receptors P2X or P2Y on the cell membrane.^12^ It is known that nucleotides are released from damaged cells in the pathogenesis of different diseases. In addition, ATP is secreted by exocytosis from many cells, including endothelial cells, red blood cells, platelets or autonomic nerves, and acts as a neurotransmitter.^13^, ^14^, ^15^ By binding to ATP, purinergic receptors trigger a chain of signalling events that affect the vital functions of many types of cells and tissues.^16^

Recent investigations have shown that extracellular ATP regulates the migrating capacity of healthy^17^, ^18^ and cancer cells,^19^, ^20^, ^21^, ^22^ suggesting the involvement of purine receptors in cancer invasion or metastasis. Moreover, ATP‐induced regulation of migration was found in stem cells and progenitor cells.^23^, ^24^, ^25^, ^26^ The capacity of stem cells to migrate to the specific tissues or organs is essential not only for normal tissue homeostasis, morphogenesis and repair, but also for creation of stem cell‐based regenerative medicines.^23^, ^24^, ^27^, ^28^

This investigation was designed to explore the impact of extracellular ATP on migration and proliferation capacity of endometrial stem cells. Our results show that ATP decreases the migration capacity of eMSCs. In addition, our results demonstrate that the cultivation of cells in the presence of ATP potently suppresses cells growth by inducing accumulation of eMSCs in G0/G1 phase.

## 2 MATERIALS AND METHODS

### 2.1 Cell cultures

Experiments have been performed on mesenchymal stem cell line (eMSCs) derived from the human endometrium. Line (2804) was obtained from Department of Intracellular Signaling and Transport of Institute of Cytology RAS (Russia).^29^ The experiments were approved by the Bioethics Committee of the Institute of Cytology of Russian Academy of Sciences and the principles of the Declaration of Helsinki. Cells exhibited properties typical for MSCs: they were adherent to plastic surface, displayed fibroblast‐like morphology, they were multipotent, showed expression of MSC‐positive surface markers CD13, CD29, CD44, CD73, CD90 and CD105 and were negative for the haematopoietic markers CD19, CD34, CD45, CD117, CD130 and HLADR (class II). Multipotency of eMSCs was tested by their ability to differentiate in other cell types, such as adipocytes and osteocytes.^29^ Additionally, the isolated eMSCs partly (over 50%) expressed the pluripotency marker SSEA‐4. Cells were grown in Dulbecco's Modified Eagle Medium (DMEM/F12) with 10% foetal bovine serum (HyClone), 1% L‐glutamine (Gibco) and 1% penicillin‐streptomycin (Gibco). The cells were cultured in culture flasks at 37°C in a humidified chamber with 5% CO_2_ and subcultured twice per week.

### 2.2 The cell migration assay

The experimental system for long‐term live cell monitoring was installed on Zeiss AxioObserver Z1 microscope equipped with ×10 objective, PlasDIC contrast and humidified microscope chamber with temperature and 5% CO_2_ control. For wound healing assay, eMSCs were seeded in 4 well plates, with pre‐installed silicone inserts, in the amount of 60 000 cells per insert. The cells were cultured for further 24 hours, then inserts were taken out, the cells were washed and cultured in full culture media containing the reagents. The wound images were taken every 1h for 48h using ‘Multidimensional acquisition’ module of Axiovision 4.8.2 software. The absolute value of the distance the cells passed was calculated as the change in the perpendicular distance between the edges of the gap for 48 hours. The value was then normalized to the 0 hour of starting measurement. Areas were calculated using ImageJ software (NIH). The percentage of area reduction or wound closure was calculated as follows: wound closure % = [(A_t=0h_‐A_t=∆h_)/A_t=0h_] × 100%, where A_t=0h_ is the area of the wound measured immediately after drug addition, while A_t=∆h_ is the area of the wound measured h (hours) after adding the drug.^30^

### 2.3 Total RNA extraction and RT‐PCR analysis

Total RNA was isolated by RNeasy Mini Kit (Qiagen). First‐strand cDNA was synthesized from 1 μg of total RNA by using 1 µg of random hexamers, 100 units of MMLV reverse transcriptase, 0.5 mmol/L dNTPs, and 1 × MMLV reaction buffer (Silex, Russia) in a total volume of 20 µL at 37°C for 1 hour. In negative control experiments, MMLV reverse transcriptase was omitted. The PCR primers were designed using the GeneRunner v5.0.59 software. To avoid false‐positive results due to genomic contamination of the samples, the primers spanned an intron at the genomic level. The primer sequences used for P2X7: 5′‐GACGCTCTGTTCCTCTGAC‐3′ (forward) and 5′‐CAGGTCTTCTGGTTCCCTTC‐3′ (reverse); expected amplicon length is 108 bp. PCR was carried out in the volume of 10 µL using 1 µL diluted (1:3) cDNA, 0.3 µmol/L of each primer, 200 µmol/L dNTPs, 2 mmol/L MgCl_2_, 1 × Hot‐Taq polymerase buffer (Silex) and 1 unit Hot‐Taq polymerase. The PCR cycling conditions were 10 minutes at 94°C; 35 cycles of 40 seconds at 94°C, 30 seconds at 60°C and 30 seconds at 72°C; 5 minutes at 72°C. About 8 µL of the PCR reaction was subjected to electrophoresis on a 6% polyacrylamide gel and then was stained with ethidium bromide and visualized by UV fluorescence.

### 2.4 Immunoblotting

Cells were washed twice with ice cold PBS and lysed in lysis buffer containing TRIS‐HCl (50 mmol/L; pH 7.4), 1% NP‐40, 150 mmol/L NaCl, 1 mmol/L EDTA, 1 mmol/L PMSF and protease inhibitor cocktail (P8340 Sigma‐Aldrich, Inc) for 15 minutes on ice. Cell lysates were cleared at 10 000 *g* for 15 minutes. Protein content was determined by the method of Bradford. Total cell lysates (30 μg) were dissolved in SDS sample buffer, separated on 8% SDS gel and transferred to nitrocellulose membrane. The membrane was incubated with a primary anti‐P2X_7_ antibody (Alomone, 1:1000) overnight and then in horseradish peroxidase‐conjugated goat anti‐rabbit IgG (Sigma A0545, 1:2000). ECL detection was performed according to the manufacturer's instructions (SuperSignal West Femto Maximum Sensitivity Substrate, Thermo Fisher Scientific Inc).

### 2.5 Calcium imaging

One day prior to the experiments, cells were seeded in 3‐cm^2^ Petri dishes containing a cover slides. After 24‐48 hours, the medium was removed, cells were washed with serum‐free medium and loaded with 4 μmol/L Fura‐2AM probe (Thermo Fisher Scientific) for 35 minutes in dark at RT. Then, cells were washed, and the cover slides were transferred into a perfusion chamber. Cell imaging was obtained using an AxioObserver.Z1 inverted microscope (Carl Zeiss MicroImaging GmbH) with a Plan‐Apo‐chromate x40/1.4 oil objective. Fura‐2AM fluorescence was excited alternately by light of 340 and 380 nm from an illuminator with a Lambda DG‐4 high‐speed wavelength switcher (Sutter Instrument Co). Analysis was performed using AxioVision 4.8.2 software (Carl Zeiss MicroImaging GmbH).

### 2.6 Immunofluorescence staining

Previously, cells were plated on a coverslip, fixed with 3.7% paraformaldehyde in phosphate‐buffered saline (PBS), permeabilized with 0.25% Tween 20 in PBS and blocked with 10% donkey serum (1 hour, at 24°C). Then, the cells were incubated with P2X_7_ antibodies, conjugated with FITC (Alomone, 1:200) at 4°C overnight. After staining, coverslips were placed with Vectashield mounting medium (Vector Laboratories) and examined using the confocal microscope Olympus FV3000 (Olympus Corporation) with ×60 oil objective.

### 2.7 FACS analysis

Adherent cells of each sample were detached with trypsin/EDTA solution and suspended in growth medium. One half of cell suspension was utilized for viability assay and the other one for cell cycle analysis by flow cytometry (FACS). Briefly, 0.05 mg/mL of propidium iodide (PI) was added to the cells and subjected to FACS analysis just after gentle mix for 30 seconds. Representative Dot Plot (FSC‐A vs PC5 5‐A) allows discriminating ‘live’ (PI‐negative) from ‘dead’ (PI‐positive) cells. The number of cells gated as PI‐negative was utilized for creating the growth curve by means of Microsoft Excel. For cell cycle analysis, saponin (0.2 mg/mL), RNAse (0.25 mg/mL) and PI (0.05 mg/mL) were added to cell suspension and incubated for 1h in dark at RT. At least 3000 events were collected for viability assay and 15 000 events for cell cycle analysis. CytoFLEX S flow cytometer (Beckman Coulter) equipped by Cytexpert software (version 2.0) was used for cytometric analysis.

### 2.8 Statistical analysis

The data are presented as the mean values of at least three independent experiments. Statistical significance was evaluated by Student's *t* test, and one‐way ANOVA with Tukey's post hoc tests for multiple comparisons, *P* < .05 were considered to be significant. Data are presented as the mean ± standard deviation (SD).

## 3 RESULTS

### 3.1 The effect of ATP on migration of human endometrial stem cells (eMSCs)

**Figure 1:**
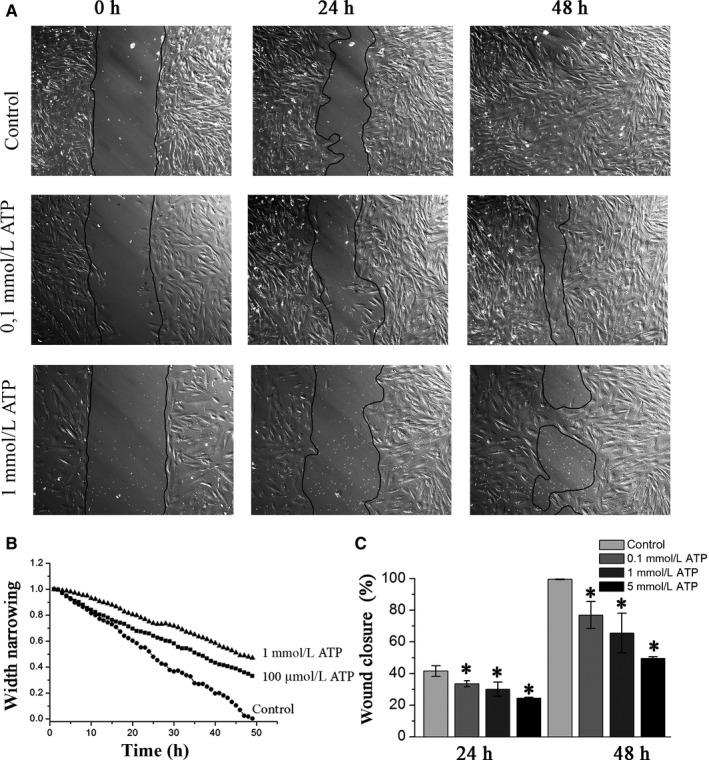
Extracellular ATP suppresses the human endometrial mesenchymal stem cell migration. A, Representative images showing the wound area at indicated time points during 48‐h incubation in culture medium with different concentration of ATP. B, The time course of wound width narrowing in the control and ATP‐treated cells culture. C, Mean wound closure in the control and ATP‐treated cell culture in four independent experiments. The data are presented as mean ± SD (n = 3), *P < .05

Now, there is growing evidence that purinergic signalling triggered by ATP regulates the migration or homing of stem cells to the specific tissues or injuries. Therefore, first of all, the wound healing assay together with time‐lapse imaging was used to establish the role of ATP in eMSCs migration. In order to avoid a possible uncontrolled release of ATP and other undesirable effects caused by cell damage, the wound in eMSCs culture was created by removal of a silicone insert as described in Material and Methods. Figure 1A demonstrates representative images illustrating the wound areas at different time points and the corresponding time course of wound healing up to 48 hours after addition of 0.1; 1.0 and 5.0 mmol/L of ATP (Figure 1B). Analysis of the wound healing area indicated that the ATP breaks the wound healing process in a dose‐dependent manner with a maximal effect obtained at 5 mmol/L ATP. Expressed as the percentage of wound closure (21), the average value of area reduction fell from 99.6 ± 0.3% in the control to 77.0 ± 8.5%, 65.6 ± 12.6% and 49.5 ± 1.1% after 48 hours of incubation with 0.1 mmol/L, 1.0 mmol/L and 5.0 mmol/L ATP, respectively (Figure 1C).

### 3.2 Expression of P2X_7_ receptor in eMSC

**Figure 2:**
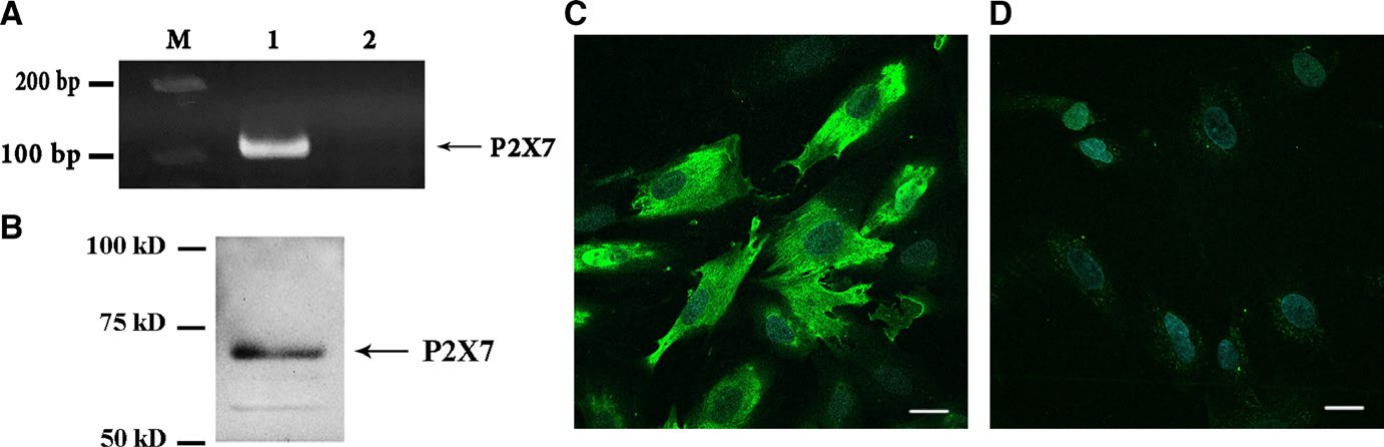
Expression of P2X_7_R in human eMSCs. A, RT‐PCR analysis of P2X_7_R expression in eMSC. 1—Primers specific for the P2X_7_R amplified a PCR product of the expected size (108 bp). 2—RT‐PCR negative control in which reverse transcriptase was omitted. M – 100 bp size marker. B, Western blot band at around 70 kD corresponding to the P2X_7_R. C, Distribution of P2X_7_R immunoreactivity in eMSCs was examined with confocal microscopy (at 60×). D, Immunoreactivity of P2X7 antibody, preincubated with the negative control antigen. Scale bar: 20 µm

Numerous studies show that ATP‐induced alterations in stem cell migration could arise from the activation of purinergic P2X_7_ receptors.^20^, ^31^, ^32^ Therefore, next, we investigated the expression of the P2X_7_R in endometrial stem cells. PCR primers were designed as indicated in Material and Methods. RT‐PCR analysis revealed the 108‐bp product corresponding to P2X_7_R transcript in eMSCs (Figure 2A). P2X_7_R protein expression was determined using Western blot analysis and polyclonal anti‐P2X_7_R antibodies. Figure 2B shows the band ~70 kD, corresponding to the P2X_7_R. Immunofluorescence confocal microscopy was used to examine the protein expression and intracellular distribution of P2X_7_R. The immunofluorescence signal was detected in the plasma membrane and in the cytoplasm of the cells labelled with the antibody recognizing the P2X_7_R (Figure 2C).

### 3.3 Ca^2+^ response in stem cells activated by purinergic stimulation

Extracellular ATP operates as an autocrine and/or paracrine signalling molecule by increasing the cytosolic Ca^2+^ concentration ([Ca^2+^]_c_) through stimulating the ionotropic P2X receptors and metabotropic P2Y receptors on the cell membrane. An increase in ([Ca^2+^]_c_) triggers the activation or modulation of many physiological processes in the cells, including migration and proliferation. Here, using calcium imaging, we conducted a series of experiments to test the participation of purine receptors in the Ca^2+^ signalling in eMSCs.

**Figure 3:**
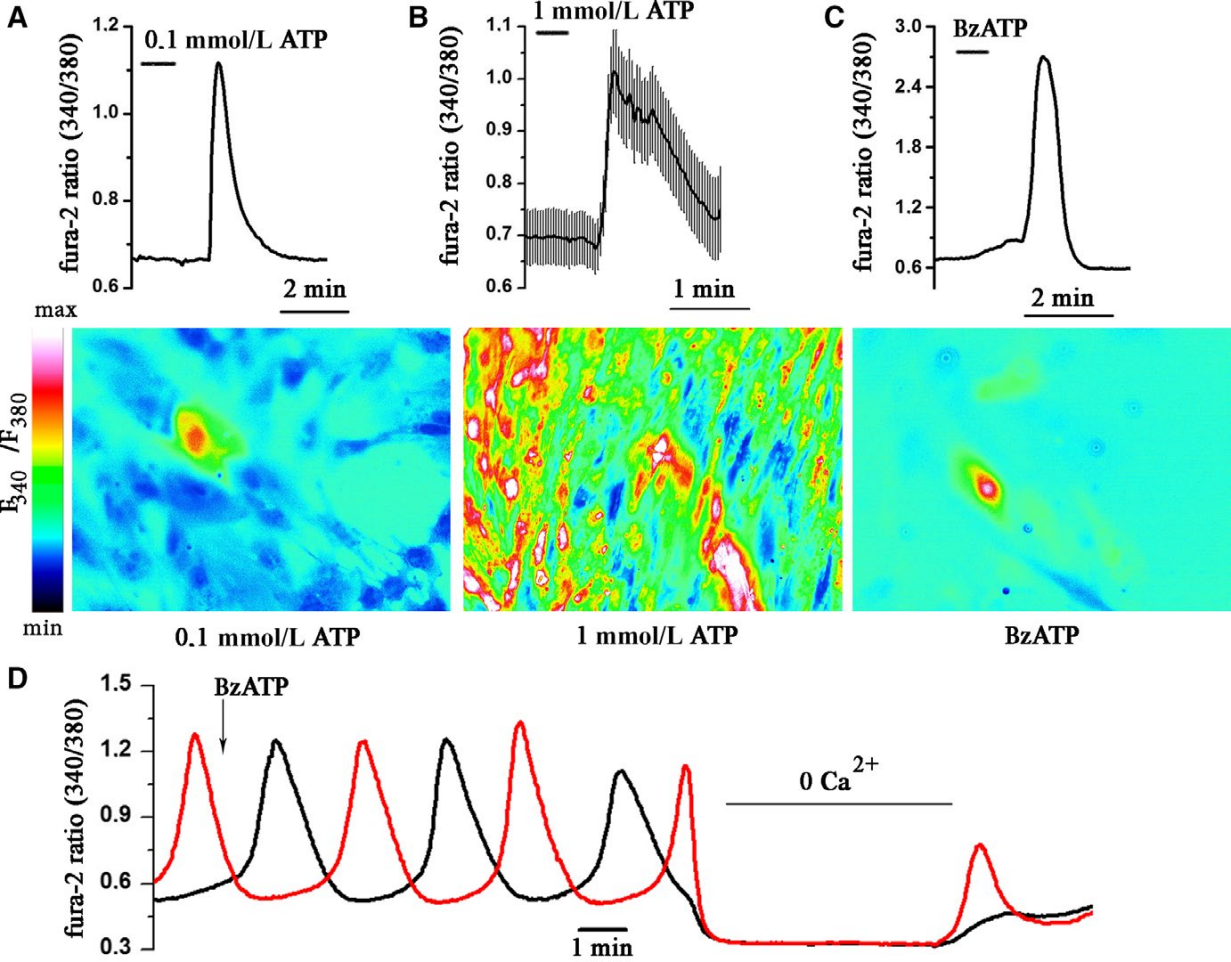
Activation of purinoceptor causes an increase in Ca^2+^ transients. A and B, Typical Ca^2+^ responses following the application of ATP and (C) BzATP. Measurements done in individual cells with the imaging system. [Ca^2+^]c is plotted versus time in control and in treated cells. B, Each point is a mean ± SD from 10 cells. The pseudocolor ratiometric calcium images correspond to the level of Ca^2+^ in cells treated with 0.1 mmol/L ATP (A), 1.0 mmol/L ATP (B) and 0.1 mmol/L BzATP (C). D, Spontaneous Ca^2+^ oscillations in eMSC cells. Removal of Ca^2+^ from the bath solution led to the disappearance of Ca^n+^ oscillations in cells

Cells loaded with dye Fura‐2AM (as described in Material and Methods) were set into the registration chamber with the flow system and analysed under a Zeiss Axiovert S100 epifluorescence inverted microscope. Images of the cells were taken every 2 seconds for 10 to 20 minutes with a Zeiss AxioCam Hrc camera. As shown in Figure 3A,B, the addition of ATP (0.1‐1.0 mmol/L) to the Ca^2+^ ‐containing bath solution results in a short‐term increase in cytoplasmic Ca^2+^ level. It is important, that no changes in ([Ca^2+^]_c_) level were observed when the cells were stimulated by ATP in Ca^2+^‐free bath solution (data not shown). This finding suggested that P2X, but not P2Y receptors mediate an increase in intracellular Ca^2+^ in eMSCs. To establish the role of P2X_7_R in ATP‐induced Ca^2+^ signalling, we used the potent P2X_7_R agonist, 20,30‐(benzoyl‐4‐benzoyl)‐ATP (BzATP). As demonstrated in Figure 3C, the addition of BzATP (0.1 mmol/L) to the bath solution caused an increase in the level of [Ca^2+^]c in eMSC. Despite the fact that stable expression of P2X_7_R was established in eMSCs, P2X_7_R‐mediated Ca^2+^ responses were observed only in several cells (Figure 3C). In general, responses to BzATP were recorded in ~8% of the cells studied, indicating that not all P2X_7_ receptors are functional and that the population of endometrial stem cells is quite heterogeneous.

In some cases, undifferentiated eMSCs displayed Ca^2+^ oscillations (Figure 3D). The oscillation frequencies differed from cell to cell, and not all of the cells demonstrated spontaneous Ca^2+^ oscillations. It should be noted that removal of Ca^2+^ from the bath solution led to the disappearance of oscillations and the recovery of Ca^2+^ to the solution led to the resumption of calcium oscillations. The addition of BzATP to the solution did not change the pattern of Ca^2+^ oscillations but could trigger the oscillations in some cells that had previously been at rest. These results indicate the existence of other (possibly store‐operated) Ca^2+^‐ conducting channels, which are supposed to be involved in Ca^2+^ oscillations in eMSCs.

### 3.4 Role of P2X_7_ receptors in eMSC migration

**Figure 4:**
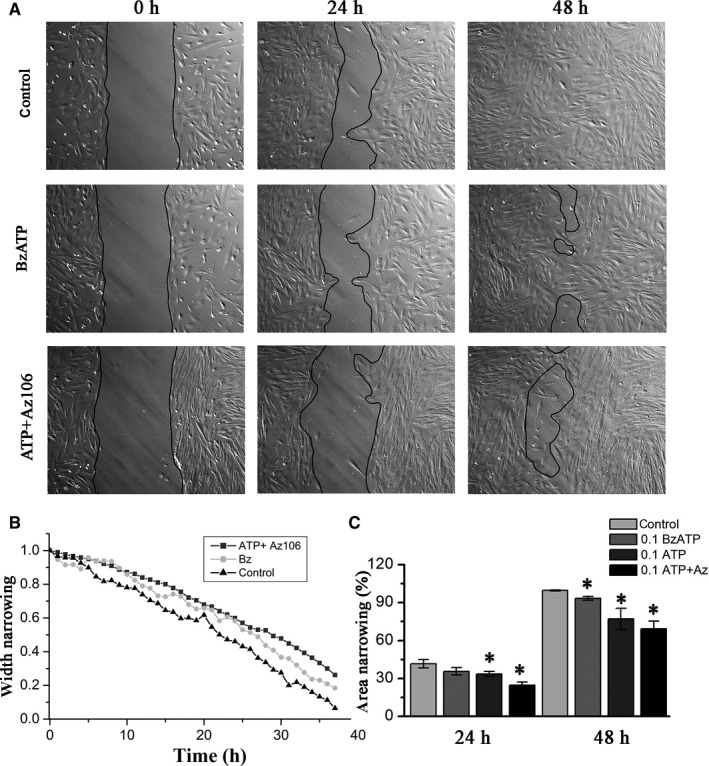
Effect of BzATP and AZ10606120 on eMSC migration. A, Representative images showing the wound area at indicated time points during 48 h in culture medium without treatment (control), after treatment with BzATP (0.1 mmol/L), after treatment with ATP (0.1 mmol/L) alone and after treatment with AZ10606120 (0.01 mmol/L) plus ATP (0.1 mmol/L). B, The time course of wound width narrowing in the control and treated cell culture. C, Mean wound closure in control, BzATP‐treated, ATP‐treated and AZ10606120‐treated cell culture in four independent experiments. The data are presented as mean ± SD (n = 4), *P < .05

To assess whether P2X_7_R affects the eMSCs migration, the cell movement was monitored for 48 hours in the presence of receptor agonist, BzATP (0.1 mmol/L) (Figure 4A,B). In addition, experiments were conducted using the selective P2X_7_ receptor inhibitor, AZ10606120 (0.01 mmol/L) in the presence and in the absence of ATP (0.1 mmol/L) in the medium. The data obtained showed that BzATP only slightly affected the movement of cells. The migration rate of eMSCs incubated in a medium with AZ10606120 in the absence of ATP had the similar values as under control conditions (data not shown). In contrast, the cell cultivation in the medium containing a both AZ10606120 and ATP resulted in suppression of cell migration (Figure 4A,B). The percentage of wound closure of eMSCs treated with BzATP was 93.0 ± 1.5% (vs control cells, *P* < .05), whereas the percentage of wound closure of cells treated with AZ10606120 in the presence of ATP was reduced to 69.4 ± 5.9%. (*P* < .05) (Figure 4C). Thus, it was established that the antagonist of P2X_7_ receptor did not prevent ATP‐induced inhibition of eMSC motility (as described in Section 3.1), indicating an insignificant role of the P2X_7_ receptor in the migration of eMSCs.

### 3.5 ATP affects the cell cycle in eMSCs

**Figure 5:**
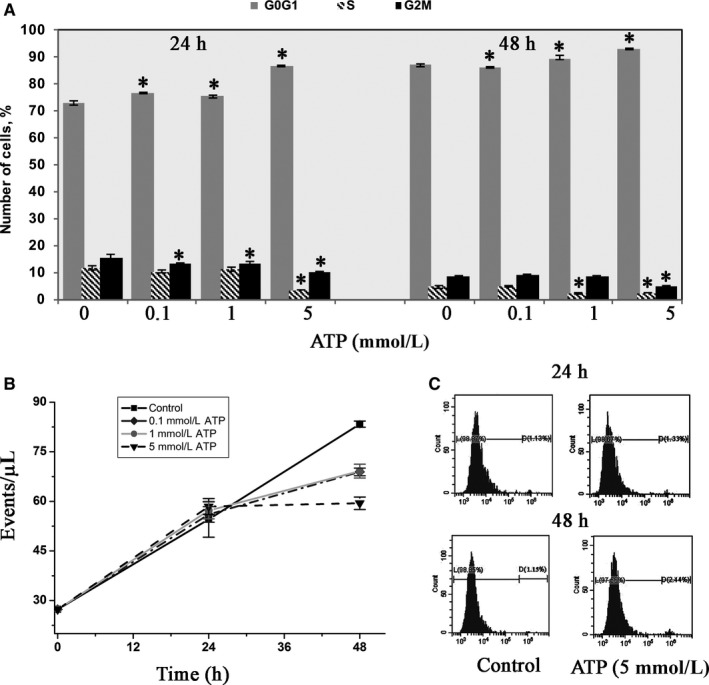
ATP treatment affects the eMSC proliferation **(**FASC analysis). A, Cell cycle distribution of eMSC cells after 24 and 48 h of cultivation in the presence and in the absence of different concentrations of ATP. ATP treatment delays G0/G1‐S transition. The data are presented as mean ± SD (n = 3), *P < .05. B, Dose‐dependent effect of ATP on cells growth. The number of PI‐negative (‘live’) cells is significantly reduced after the 48‐h incubation with ATP (see ‘Materials and Methods’). Growth curves of control (1, filled circles) and treated with 0.1, 1.0 and 5.0 mmol/L ATP eMSCs (2, filled rhombus; 3, square and 4, triangles, respectively). The data are presented as mean ± SD (n = 3), *P < .05. C, Representative histograms of eMSC distribution after 5 mmol/L ATP treatment; viability assay with PI staining. The percentage of dead cells after 24 (upper panel) and 48 h (lower panel) of cells incubation with ATP

Next, we assumed that ATP‐mediated inhibition of cell migration might occur because of changes in the proliferation rate of eMSC culture. To test this hypothesis, we monitored the ATP‐treated cell distribution on different phases of the cell cycle using flow cytometry DNA analysis. The FACS analysis revealed the dose‐dependent cells accumulation in G1 phase with simultaneous decrease in S and G2/M phases after ATP treatment (Figure 5A). It is important to note that the strength of the ATP effect was depended on drug concentration and exposure time. The FACS analysis showed a slight increase in the number of eMSC in the G0/G1 phases and a simultaneous their decrease in the S‐G2M phases after 24 hours of cell incubations with 0.1 and 1.0 mmol/L ATP. However, after addition of 5.0 mmol/L ATP, there was a significant inhibition on cell cycle phase transition and eMSC accumulation in the G0/G1 phases (86.4 ± 0.4% vs 72.7 ± 0.6% in control). The more pronounced ATP effect was observed after long‐term cells cultivation. The percentage of cell population in S phase fell by half: from 4.7 ± 0.3% in control to 2.2 ± 0.1% and 2.4 ± 0.1% after 48 hours cells incubation with 1.0 mmol/L and 5.0 mmol/L ATP (Figure 5A).

In addition, we found a strong growth inhibition of eMSCs after 48 hours of treatment with all selected concentration of ATP (Figure 5B). Counting the number of PI‐negative cells reveals: 59.4 ± 1.8 (cells/μL); 69.1 ± 2.0 cells/μL) and 68.9 ± 1.1 (cells/μL) after 48 hours of incubation with 5.0; 1.0 and 0.1 mmol/L ATP vs 83.3 ± 0.9 (cells/μL) in control. In parallel with cell cycle analysis, we estimated the cell viability of eMSCs by PI staining. We found that the percentage of viable cells in population did not actually change after 48 hours of treatment with ATP (2.94 ± 0.4% died cells in 5.0 mmol/L ATP vs 1.91 ± 0.57% died cells in control) (Figure 5C). In general, the data show that ATP, applied to eMSCs does not affect the cell survival but causes a cell cycle delay at G1 phase and a decrease in cell growth rate.

## 4 DISCUSSION

Stem cells exist in all organs, providing the replacement of damaged and sick tissues. Damaged tissues and organs release numerous compounds, including intracellular ATP, which are triggers many cellular processes. ATP can be emitted by exocytosis, by traumatic cell lysis, or by passive leakage from damaged cells.^33^ In healthy tissues, the extracellular ATP concentration is adjusted by ecto‐nucleotidases, such as ecto‐ATP/ADPase (CD39).^34^ In damaged tissues, the impaired activity of CD39 and the release of ATP can result in accumulation of extracellular ATP.^35^ Extracellular ATP through the activation of P2X and P2Y purinergic receptors affects the migration, proliferation and differentiation of many cell types, including stem cells.

Here, we addressed the impact of ATP on eMSCs migration. It was determined that millimolar concentrations of ATP potently inhibited the migration of eMSCs in a dose‐dependent manner. Incubation of cells with 5.0 mmol/L ATP for 48 hours reduced the migration rate by ~50%. Recent studies have demonstrated that ATP has a significant effect on migration of different types of cells. However, as a rule, the application of ATP led to the acceleration, rather than inhibition of migration of stem and cancer cells, indicating that MSCs from various sources exhibit different biological properties, which may be relevant for clinical using of distinct types of MSCs. For example, cell movement examined by transwell migration assay showed that ATP treatment accelerates bone marrow‐derived mesenchymal stem cells (BM‐MSC) migration.^27^, ^36^ Activation of purinoceptors also promoted the migration of glioma cells and pancreatic ductal adenocarcinoma cells.^20^, ^32^

Recently, there has been some interest in P2X_7_ receptor, which is triggered by high concentrations of ATP (>100 μmol/L)^27^, ^36^ and activates Ca^2+^ influx in cells. The literature data report the expression and involvement of the P2X_7_R in dental pulp‐derived mesenchymal stem cell (DP‐MSC) migration.^31^ In our study, PCR and immunoblot analysis revealed the expression of P2X_7_ mRNA and protein in endometrial stem cells. Images of cells labelled with an antibody that recognizes P2X_7_R showed the stable immunoreactivity. Nevertheless, P2X_7_R ‐mediated Ca^2+^ response was obtained in a small amount of eMSCs, suggesting that only a few cells express a functional receptor. Moreover, in our experiments, neither activation nor inhibition of P2X_7_R activity affected the rate of wound healing, indicating that P2X_7_R does not play a significant role in eMSC migration. Beyond the P2X receptors, mammalian cells can also express a classical G‐protein‐coupled receptors, P2YR.^12^ ATP‐induced stimulation of P2YR triggers activation of phospholipase C and production of inositol triphosphate (IP_3_) followed by IP3R activation and Ca^2+^ release from the intracellular stores. Measurement of Ca^2+^ concentrations in calcium‐free solutions revealed no Ca^2+^ rise in eMSCs after stimulation with ATP, indicating no functional activity of P2Y receptors in cells.

According to numerous reports, ATP can affect the proliferation and viability of some cell types, including stem cells.^27^, ^36^, ^37^, ^38^ Here, we show that ATP in a dose‐related manner suppressed the eMSCs proliferation, while the viability of cells was not altered throughout the period of their cultivation with ATP. Our data are consistent with the results reported for human bone marrow mesenchymal stem cells,^37^ human endometrial stromal cells^39^ and also neonatal rat cardiac fibroblasts,^40^ which show that ATP regulates MSCs proliferation activity and probably is one of the factors determining the cell fate.

Many reproductive dysfunctions are connected with disturbed endometrial proliferation. The eMSCs are necessary for endometrial recovery during each menstrual cycle. Accordingly, decrease in the number of cells or disturbance of their functions can lead to the formation of thin endometrium that is unable to maintain gestation.^41^ The potential of endometrial MSCs offers a broad perspective in the treatment of such diseases. Besides the eMSCs may play an important role in the pathophysiology of such diseases as human adenomyosis or endometriosis that are cause of infertility.^42^, ^43^

In conclusion, our results indicate that ATP suppresses the proliferation of eMSCs that accompanied by cells arrest in G1 phase of cell cycle. We believe that ATP‐induced suppression in eMSC proliferation led to decrease in the migration capacity of these cells; however, the exact mechanism by which ATP acts on eMSCs needs further investigation. We are convinced that monitoring of processes associated with ATP‐dependent regulation of cell migration and proliferation will allow to significantly improve the regenerative potential of eMSCs for use in tissue engineering and regenerative medicine.

## CONFLICT OF INTEREST

The authors declare no conflict of interest.

## AUTHORS' CONTRIBUTIONS

S. S., designed research, performed the cell staining and Ca^2+^ imaging, analysed data and wrote the paper; AS, performed cell cycle analysis and analysis of cell viability; IV performed PCR and Western blot analysis, NP performed the cell cultures; MS, performed the cell migration research; YN, developed the study concept, supervised the research team. All authors were involved in the manuscript preparation and editing.

## Data Availability

The data that support the results of this investigation are accessible from the corresponding author upon request.
